# 417. Impact of Post-acute Sequelae of COVID-19 (PASC) on Healthcare Workers (HCW) in NYC Public Hospital in the South Bronx

**DOI:** 10.1093/ofid/ofad500.487

**Published:** 2023-11-27

**Authors:** Chee Yao Lim, Jorge Gutierrez, Alberto Romero Garcia, Pinal Patel, Kenneth Johan, Israel Duran, Jeffery Evans, Anna Riordan, Inderpreet Singh, Masood A Shariff, Rabia Ashraf, Moiz Kasubhai, Nehad Shabarek, Vihren Dimitrov, Vidya Menon

**Affiliations:** NYCHHC/Lincoln, New York, NY; NYCHHC/Lincoln, New York, NY; NYCHHC/Lincoln, New York, NY; Lincoln Hospital, Bronx, New York; NYCHHC/Lincoln, New York, NY; NYCHHC/Lincoln, New York, NY; NYCHHC/Lincoln, New York, NY; NYCHHC/Lincoln, New York, NY; NYCHHC/Lincoln, New York, NY; NYC HHC Lincoln, Bronx, New York; NYCHHC/Lincoln, New York, NY; NYCHHC/Lincoln, New York, NY; Lincoln medical center, NYC, New York; NYCHHC/Lincoln, New York, NY; Lincoln Medical Center, New York, New York

## Abstract

**Background:**

COVID-19 infection is known to cause long-term sequelae with impact on multiple systems. HCW are at increased risk for COVID-19 exposure and infection. The aim of our study was to assess the burden of PASC among HCW in NYC public hospital which was at the epicenter of the pandemic.

**Methods:**

In this single-center, cross-sectional study, HCW with previous COVID-19 infection were administered an anonymous voluntary survey to evaluate PASC, and its impact on their quality of life (QoL) and mental health. We used CDC COVID-19 variant data tracker to identify predominant variants during the infection. Average number of symptoms/signs, severity, and changes in quality of life were analyzed using JASP.

**Figure:**
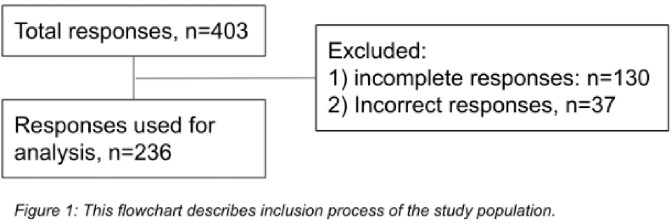

Inclusion Process of Study Population

**Results:**

Out of the 236 subjects, 144 (61%) had PASC, with 78% having 2 or more symptoms/signs with fatigue being the most common (40%) [Fig 2]. Women had significantly more symptoms than men (2.37 vs 1.26, p=0.005). The mean number of symptoms was comparable among age groups. Average numbers of symptoms were significantly higher among patients who were involved in direct patient care vs indirect patient care. Patients who had reinfection had more symptoms than patients without (2.52 vs 1.76, p=0.02). Among the variants of concern, wild and alpha variants were associated with a higher number of symptoms (p=0.01). Cognitive dysfunction and mental illness (depression/anxiety) were observed to be significantly worsened following COVID-19 infection. PASC adversely affected activities of daily living and disrupted lifestyle.

**Figure:**
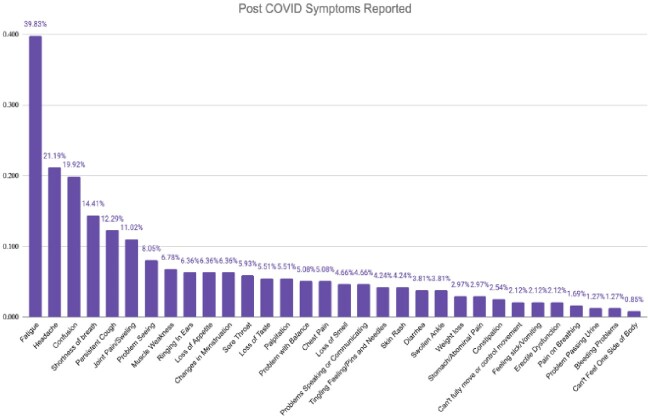

Breakdown of symptoms/signs according to frequency

Subject Baseline Characteristics

**Figure:**
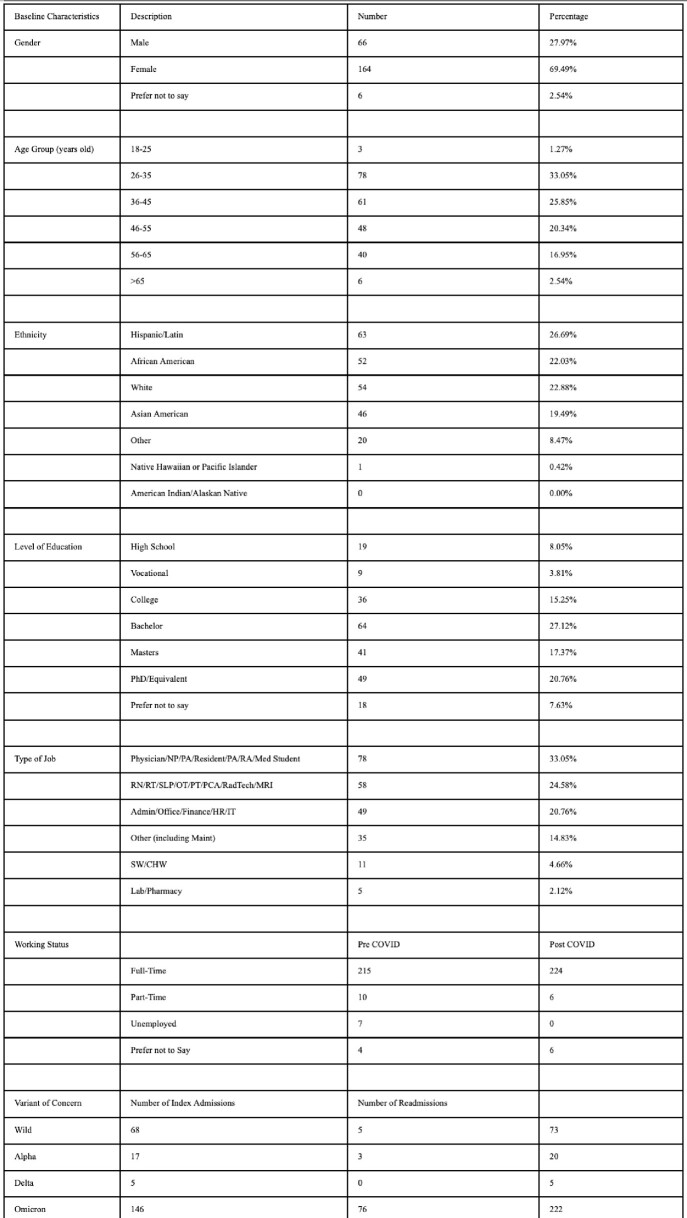

**Figure:**
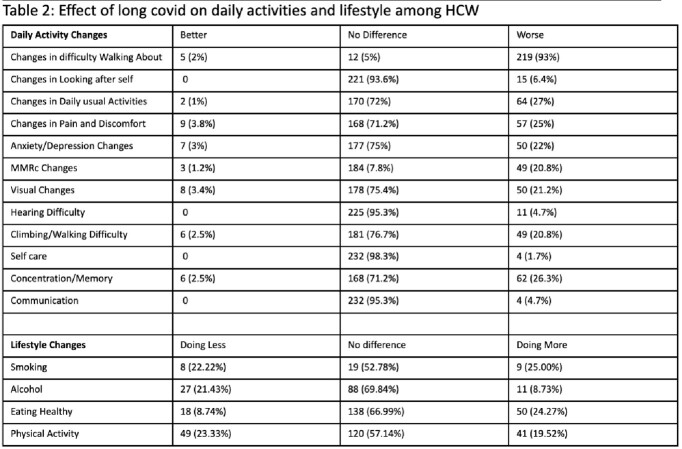

**Conclusion:**

Significant burden of PASC was observed in our healthcare community. The short- and long-term consequences of PASC for HCW could severely affect healthcare services and it is crucial to conduct research and allocate resources to mitigate and overcome these issues.

**Disclosures:**

**All Authors**: No reported disclosures

